# Behavior of rat bone marrow stem cells on titanium surfaces modified by laser-beam and deposition of calcium phosphate

**DOI:** 10.1007/s10856-021-06528-4

**Published:** 2021-05-17

**Authors:** F. Florian, F. P. S. Guastaldi, M. A. Cominotte, L. C. Pires, A. C. Guastaldi, J. A. Cirelli

**Affiliations:** 1 Departament of Morphology – Anatomy, Araraquara Dental School, UNESP, Araraquara, SP Brazil; 2 Department of Diagnosis and Surgery, Araraquara Dental School, UNESP, Araraquara, SP Brazil; 3 Department of Oral and Maxillofacial Surgery, Massachusetts General Hospital, Harvard School of Dental Medicine, Boston, MA USA; 4 Department of Physical Chemistry, Institute of Chemistry of Araraquara, UNESP, Araraquara, SP Brazil

## Abstract

**Objectives:**

The aim of this study was to evaluate the behavior of rat bone marrow stem cells seeded on a Ti-15Mo alloy surface modified by laser-beam irradiation followed by calcium phosphate deposition.

**Materials and methods:**

A total of four groups were evaluated: polished commercially pure titanium (cpTi): Ti-P; laser irradiation + calcium phosphate deposition on cpTi: Ti-LCP; polished Ti-15Mo alloy: Ti15Mo-P; and laser irradiation + calcium phosphate deposition on Ti-15Mo alloy: Ti15Mo-LCP. Before and after laser irradiation and calcium phosphate deposition on the surfaces, physicochemical and morphological analyses were performed: Scanning Electron Microscopy (SEM) and Energy Dispersive Spectroscopy (EDX). The wettability of the samples was evaluated by contact angle measurement. In addition, the behavior of osteoblast-like cells to these surfaces was evaluated for cell morphology, adhesion, proliferation and viability, evaluation of alkaline phosphatase formation and gene expression of osteogenesis markers.

**Results:**

Surfaces wet-abrade with grit paper (P) showed oriented groves, while the laser irradiation and calcium phosphate deposition (LCP) produced porosity on both cpTi and Ti15Mo alloy groups with deposits of hydroxyapatite (HA) crystals (SEM). EDX showed no contamination after surface modification in both metal samples. A complete wetting was observed for both LCP groups, whereas P surfaces exhibited high degree of hydrophobicity. There was a statistical difference in the intragroup comparison of proliferation and viability (*p* < 0.05). The ALP activity showed higher values in the Ti15Mo alloy at 10 days of culture. The gene expression of bone related molecules did not present significant differences at 7 and 14 days among different metals and surface treatments.

**Conclusion:**

Ti15-Mo seems to be an alternative alloy to cpTi for dental implants. Surface treatment by laser irradiation followed by phosphate deposition seems to positively interact with bone cells.

**Clinical relevance:**

Ti-15Mo alloy surface modified by laser-beam irradiation followed by calcium phosphate deposition may improve and accelerate the osseointegration process of dental implants.

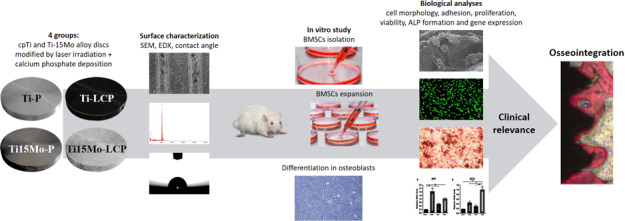

## Introduction

Commercially pure titanium (cpTi) and Ti-6Al-4V alloy are the most commonly materials used for the manufacture of dental implants due to its biocompatibility, biomechanical properties capable of supporting masticatory loads [1–4], and because it allows osseointegration, a direct contact between bone and the surface of the implant [5–7]. However, they exhibit higher elasticity modulus in comparison to both human cortical and cancellous bone [4, 8]. Furthermore, it has been shown that ions released by the Ti-6Al-4V alloy can be toxic [9, 10]. Current research tends to address the development of new materials with mechanical properties similar to bone to favor healing at the implant/bone interface and composed of biocompatible and nontoxic elements. The development of Ti alloys with low elasticity modulus and nontoxic elements is desirable and advantageous [1, 2, 4, 8, 11]. Moreover, recent studies have shown that β phase Ti alloys provides suitable mechanical properties for dental implants [12, 13]. Molybdenum (Mo) is one of the nontoxic elements used in the development of Ti alloys. In addition, previous studies have shown that the higher the concentration of Mo (15 and 20%) the greater the retention of β phase [1, 2, 4, 8, 11].

One of the challenges in the field of implant dentistry is to accelerate the growth of bone tissue around the implant surface shortening the overall time of treatment with both predictability and longevity. Thus, methods to reduce the time and increase the quality of osseointegration have focused on modification of the main properties of implants surface (mechanical, topographical or physicochemical) [1, 4, 5, 14]. The development and the improvement of the materials used for manufacturing dental implants aims at mechanical resistance, including corrosion and wear prevention, seeking to develop increasingly effective implants.

The irradiation of the surface by high power laser-beam leads to a nanostructured and tridimensional surface, with enhanced roughness, resistance to corrosion and wear, absence of contaminants on the surface, an increased Ti oxide layer by ablation phenomenon. In addition, this method can be carried out in a controlled and reproducible manner [12, 15–19]. Hydroxyapatite (HA) is the main mineral component of the bones, being the calcium-phosphate (CaP) compound more studied as bioceramic. The association of HA biocompatibility and the properties of Ti or its alloys drive to a potential for biomedical applications. Substrates containing CaP coat exhibit faster biological fixation between implants and bone tissue when compared to those without CaP [20, 21].

Osteoblastic progenitor stem cells play essential roles in the osseointegration process, including cells recruitment, adhesion, proliferation, differentiation and deposition of mineralized matrix [22]. The proliferation phase might be influenced by nanoporous topography, through selective adhesion of osteoblastic progenitor cells on the surface, which can accelerate the bone healing process around implants [23, 24]. Therefore, when considering osseointegration, decreased osteoblast cell number and proliferation have been associated with negative results [25–27]. Several studies have been conducted to investigate various implant surfaces nanostructures and their influence on the cell proliferation phase, in contrast to other scopes of surface structures dimensions [27–30]. However, the ideal implant surface nanostructure coating for osteoblasts proliferation is yet to be established. The central focus of implant development is to minimize bacterial adhesion while promoting the recruitment, adhesion, and proliferation of osteogenic cells, ensuring the long-term success of implants [30, 31].

Therefore, the aim of this study was to perform a physicochemical and morphological characterization of a Ti-15Mo alloy surface modified by laser-beam irradiation with additional deposition of calcium phosphate and to evaluate in vitro the biological behavior of rat bone marrow stem primary cells on different surfaces, evaluating cell morphology, adhesion, proliferation and viability, and the potential of the surface to stimulate osteogenesis.

## Materials and methods

### Samples preparation and surface modification

CpTi discs (area, 78.5 mm^2^; thickness, 2 mm; Titanews Barueri, SP, Brazil) and Ti-15Mo alloy discs (area, 78.5 mm^2^; thickness, 2 mm; developed by the Biomaterials Group, Institute of Chemistry of Araraquara, UNESP, SP, Brazil) were used in the present study. The discs were mechanically polished under water cooling with 100 to 600-grit grinding paper. Subsequently, the discs were ultrasonically cleaned (Ultramet 2003 Sonic Cleane, Buehler), immersed for 15 min in acetone (Sigma Aldrich, St Louis, MO), and washed with deionized water and 100% ethanol (Sigma Aldrich, St Louis, MO). The discs were washed again for 15 min in deionized water and dried at room temperature (25 °C).

Half of the disks had their surface treated with laser-beam irradiation using a pulsed laser Yb:YAG Omni Mark 20F (Pulsed Ytterbium Fiber Laser, Ominitek Tecnologia Ltda, SP, Brazil). The following laser-beam parameters were used: fluency (density power inside irradiated surface) 1,9 J/cm^2^, scanning speed 0–200 mm/s, pulse frequency 20–35 kHz and average exposure area 14 mm^2^.

The deposition of the calcium phosphate was performed after the laser irradiation, through the biomimetic method elsewhere described [32] using modified biomimetic solution, composed of NaCl, NaHCO_3_, K_2_HPO_4_, HCl, CaCl_2_.2H_2_O and TRIS, aiming the deposition of several types of apatites. A total of four groups were obtained: 1) polished cpTi (Ti-P), 2) laser irradiated + calcium phosphate deposition cpTi (Ti-LCP), 3) polished Ti-15Mo alloy (Ti15Mo-P) and 4) laser irradiated + calcium phosphate deposition Ti-15Mo alloy (Ti15Mo-LCP). All samples were sent to the Brazilian Sterilization Company (Jarinu, SP), for sterilization with gamma radiation.

### Physicochemical and morphological characterization

The morphology of the studied surfaces was analyzed by High Resolution Scanning Electron Microscopy (FEG-SEM; JEOL, model 7500F equipped with an Oxford Link ISIS 300 EDX), and the Energy Dispersive Spectroscopy (EDX) was used to check the chemical elements or detect any contamination of the materials.

The wettability of the samples was evaluated by contact angle measurement using a contact angle tester OCA-15 (Dataphysics, Germany). Drops of distilled water were delivered onto the specimen surface by a syringe giving the same drop size. The contact angle was measured after 20 s and repeated 3 times for each sample.

### In vitro study

The present study evaluated the behavior of rat bone marrow stem primary cells seeded in samples from the four different groups previously described. For all tests, 2 × 10^5^ cells were seeded on the discs surface in 50 µL of culture medium and after 1 h (adhesion initial period) the volume of medium was supplemented to a final volume of 1000 µL. Cells were grown in individual wells in 24 well-plates, incubated in medium α-MEM, supplemented with 10% fetal bovine serum, antibiotics (Penicillin 100 IU/mL and Streptomycin 100 µg/mL - Sigma Aldrich, St Louis, MO), 2 mmol/L of ascorbic acid and 10 mM/L β-glycerophosphate, in a humidified atmosphere of 5% CO_2_ at 37 °C. For all the analyses, three independent experiments were performed in triplicate.

### Rat bone marrow stem cell (rBMSC) primary culture

Rat bone marrow stem cell were isolated from femur obtained from 15 to 21-day-old rat Rattus Norvegicus Holtzman by sequential procedure approved by the Ethical Committee for Animal Experimentation from the School of Dentistry at Araraquara and used for all methods described in this study (Protocol 34/2014), in accordance with the EU Directive 2010/63/EU for animal experiments.

The bone marrow contents were centrifuged and resuspended in 10 mL of α-MEM, supplemented with 10% fetal bovine serum, penicillin 100 IU/mL and streptomycin 100 µg/mL (Sigma Aldrich, St Louis, MO). They were seeded in 100 mm culture dishes and cultured in a humidified atmosphere of 5% CO_2_ at 37 °C. After 24 h were made the exchange of the culture medium for the removal of cells not adhered and after that, exchange the medium 3 in 3 days, until the cells reach a confluence ~80%, which were frozen in FBS with 10% dimethysulfoxide (DMSO) or following the continuity of the experiment.

### Cellular morphology

The effects of the different surfaces on cellular morphology and spreading was evaluated by SEM at 3 and 8 days. Cells were cultured on discs and then fixed with glutaraldehyde 2% (Sigma) in pure α-MEM (Gibco) and glutaraldehyde 2% in 0.1 M cacodylate buffer (Sigma), washed in PBS and dehydrated in increasing concentrations of ethanol. After drying in a vacuum desiccator, samples received gold deposition by SCD 050 Sputter (Bal-Tec) at 50 s, current 40 mA with 12 nm coverage. The stages of spreading were determined in accordance with a proposal published elsewhere [33] and the cell were classified at stage 1 (round cells), 2 (round cells with filopodia), 3 (cells with cytoplasmic webbing), and 4 (well flattened cells). Imaging was performed by SEM Magellan 400L (FEI).

### Cellular proliferation

Cellular proliferation was evaluated by direct fluorescence with Alexa Fluor 488-conjugated phalloidin (Molecular Probes, Eugene, OR, USA), which labels ubiquitous actin cytoskeleton, and 4’,6-diamidino-2-phenylindole, dihydrochloride (DAPI, Molecular Probes), for nuclear stain. Cellular proliferation and spreading were evaluated at 3, 8 and 15 days.

### Cellular viability

AlamarBlue® is an important redox indicator that is used to evaluate metabolic function and cellular health. The effect of different surfaces and alloys on cell viability was evaluated by using the reagent in periods of 3, 9, 15, 18 and 21 days of culture cells. The cells seeded on the discs were incubated containing 500 µL of work solution (α-MEM with 10% FBS, Penicillin 100 IU/mL, Streptomycin 100 µg/mL and 10% AlamarBlue®) and as negative control the AlamarBlue® work solution. After 4 h of incubation period, 150 µL of each sample were collected, transferred to a 96 well-plate and read on a spectrophotometer at wavelengths of 570 and 600 nm. The number of viable cells is associated with the die reduction level and is expressed as percentage of reduction of AlamarBlue®, according with the manufacturer´s instruction.

### Production of alkaline phosphatase activity

After 10 and 14 days, the cells seeded on Ti discs underwent lysis with 300 μL/well of 1% Triton X-100 (Sigma). The lysates were quantified by modified Lowry method with a Total Protein Kit (Sigma Aldrich, St Louis, MO, USA). ALP activity was assessed by measurement of the release of thymolphthalein from thymolphthalein monophosphate in a commercial test kit (Labtest Diagnostica SA, Belo Horizonte MG, Brazil), following the manufacturer´s instructions.

### Gene expression of bone markers

Quantitative real-time PCR (qPCR) was performed to analyze the expression levels of the genes encoding the transcription factors runt-related transcription factor 2 (RUNX2), alkaline phosphatase (ALPL), osteocalcin/bone gamma-carboxyglutamic acid protein (BGLAP) by cells in response to both cpTi and Ti-15Mo different surfaces, at 7 and 14 days timepoints.

Total RNA was extracted and quantified at each incubation time by using RNeasy Mini kit (Qiagen, Valencia, CA, USA). Each sample was quantified by photometry (Eppendorf, Hamburg, Germany) and considered acceptable when the 260/280 nm absorbance ratio was higher than 1.8. The cDNA was synthesized from each RNA sample by the High Capacity Reverse Transcription Kit (Applied Biosystems, Foster City, CA, USA) with 300 ng of total RNA in a 20 μL reaction.

The qPCR reactions for RUNX2, ALPL, BGLAP were performed with inventoried assays (Applied Biosystems) (Table 1), cDNA, and Taqman Universal Master Mix (Applied Biosystems). Reactions were run in a Step One Plus real-time thermocycler (Applied Biosystems). Relative mRNA expression was determined using the delta-delta CT method, with the gene encoding GAPDH as the reference gene.

**Table 1:** List of inventoried assays used to perform the quantitative real-time PCR (qPCR) analysis

Primers	Code	Amplicon length	Gene ID
**ALPL**	Rn 01516028_m1	68	25586
**BGLAP**	Rn 00566386_g1	104	25295
**RUNX2**	Rn_01512298_m1	86	367218

### Statistical analysis

All tests were performed using Prism 5.0 (GraphPad Software, San Diego, CA, USA). For comparisons of three or more groups, the Kruskal–Wallis non-parametric data test with Dunn’s post-test was used. For comparisons between two groups, the non-parametric Mann–Whitney test was used. The level of significance was set to be 5%.

## Results

### Physicochemical and morphological characterization

**Fig. 1:**
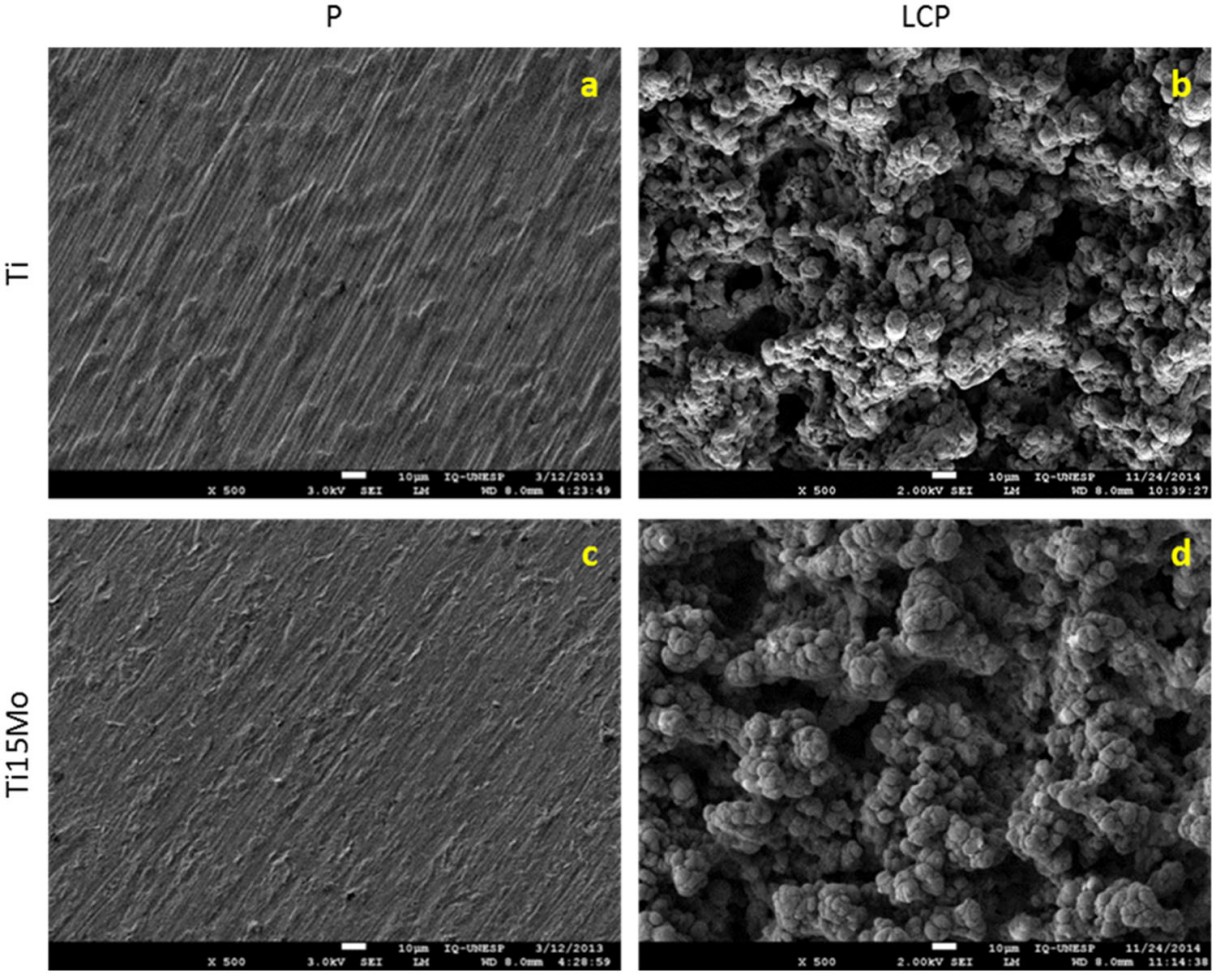
FEG-SEM of Ti and Ti15Mo samples (1.000x). (**a**) Ti-P; (**b**) Ti-LCP; (**c**) Ti15Mo-P; (**d**) Ti15Mo-LCP

**Fig. 2:**
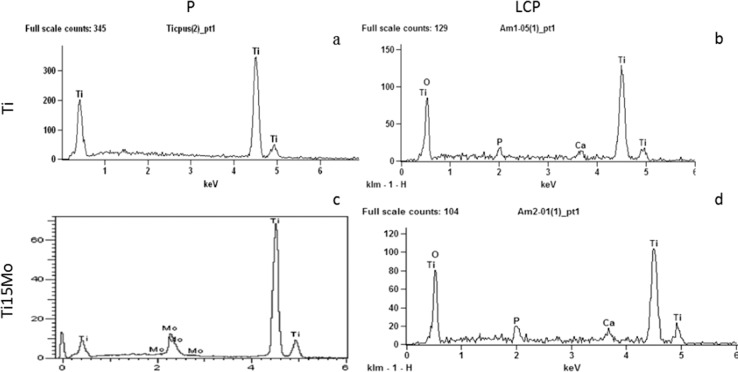
EDX of Ti and Ti15Mo samples. (**a**) Ti-P; (**b**) Ti-LCP; (**c**) Ti15Mo-P; (**d**) Ti15Mo-LCP

Surface topography analysis of laser irradiated and calcium phosphate deposition (Ti-LCP and Ti15Mo-LCP) and polished (Ti-P and Ti15Mo-P) samples were carried out using FEG-SEM. Laser-beam irradiation and the calcium phosphate deposition promoted clear differences in the surface morphology compared to polished substrates. Surfaces wet-abrade with grit paper (P) showed oriented groves (Fig. 1a, c) compatible with the procedure, while the laser irradiation and calcium phosphate deposition (LCP) (Fig. 1b, d) produced porosity on both cpTi and Ti15Mo groups with deposits of HA crystals. The EDX microanalysis was carried for all samples and the chemical elements was assessed (Fig. 2). The elements Ti (a) and Ti+Mo (c) were detected for both cpTi and Ti15Mo polished groups, respectively, and Ca+P+O enrichment was observed for cpTi and Ti15Mo after laser-beam irradiation and calcium phosphate deposition. Furthermore, the EDX analysis did not show contamination, in both Ti-LCP and Ti15Mo-LCP groups.

The contact angle values (0° ≤ Ɵ ≤ 90°) from cpTi and Ti-15Mo samples were measured to evaluate the influence of polished and laser-beam with calcium phosphate deposition treatment on surface wettability. A complete wetting was observed for laser irradiated disks, whereas polished surfaces exhibited high degree of hydrophobicity. The mean as well as the standard deviation (SD) for each sample can be seen in Table 2.

**Table 2:** Mean and standard deviation (SD) contact angle measurements of Ti and Ti15Mo groups after polishing (P) or laser-beam irradiation and calcium phosphate deposition (LCP)

Samples	Mean	SD
**Ti-P**	72.4°	2.40°
**Ti-LCP**	0°	–
**Ti15Mo-P**	72.3°	3.42°
**Ti15Mo-LCP**	0°	–

### Cellular morphology

The RBMSC cells grown on the surfaces of both Ti-P and Ti15Mo-P discs showed similar cellular morphology and spreading within 3 days. In this timepoint, the cells were in the initial stage of proliferation, rounded in shape and adhered in the surface. Some of them showed an elongated shape, emitting their first extensions, called filopodia. At 8 days, the cells were in greater number and presented more prolongations, forming agglomerates.

**Fig. 3:**
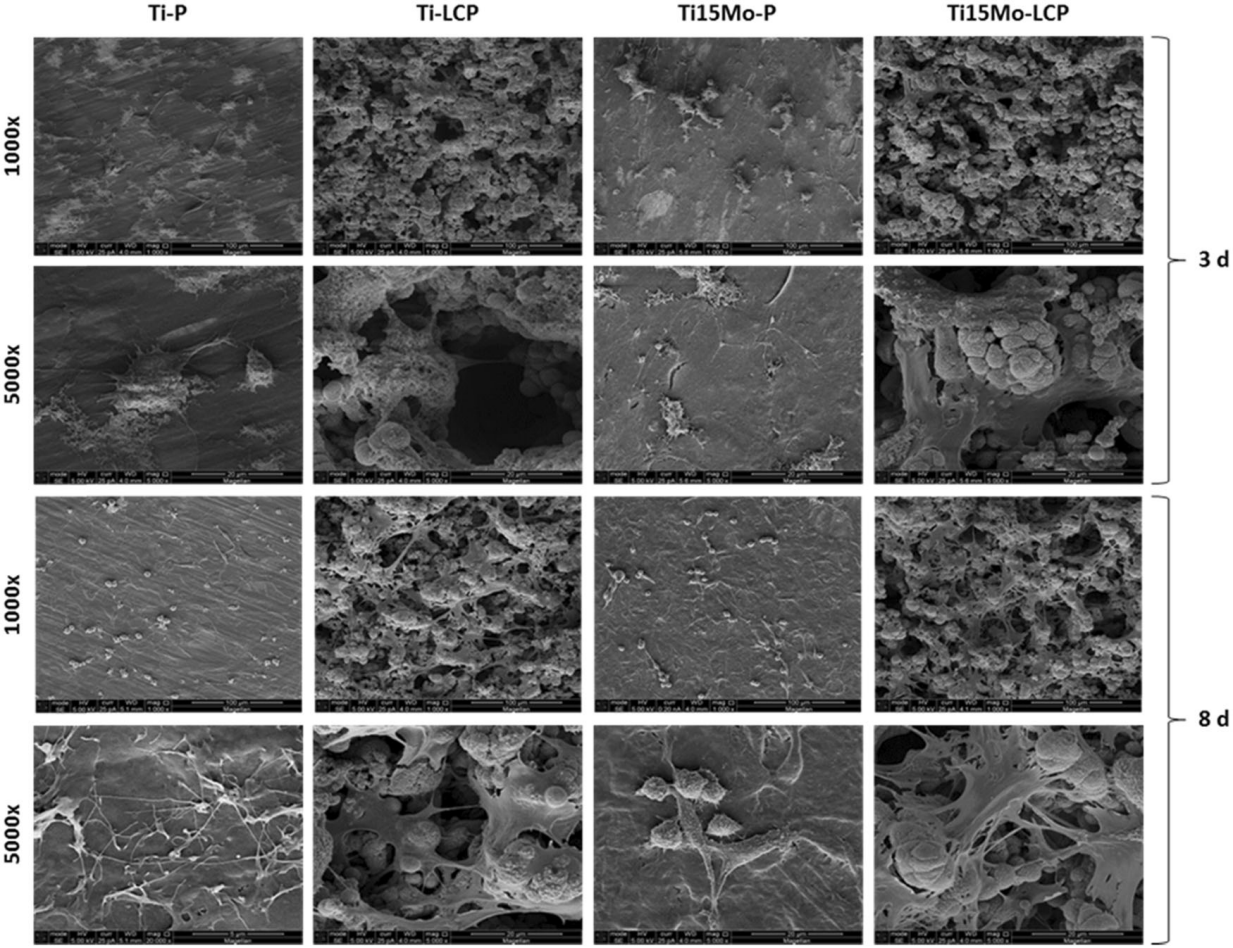
Cellular morphology observed by FEG-SEM (1000X and 5000X) at 3 and 8 days

On the other hand, LCP treatment appeared to favor cell growth in comparison to polished groups, independently of the metal (cpTi or Ti15Mo). There was a larger area of cellular contact created by the structure of laser irradiation and calcium phosphate deposition in which the cellular extensions united over time, forming a three-dimensional cellular structure (Fig. 3).

### Cellular proliferation

Direct fluorescence microscopy identified the cell morphology using fluorescence dye Alexa Fluor 488 and DAPI (4’,6-diamidino-2-phenylindole) coloring the cytoplasm (green) and cell nucleus (blue), respectively. Cells cultured on both Ti-LCP and Ti15Mo-LCP groups at 3 days, were shown in the early stages of cell adhesion, with a small, polygonal shape and with difficult visualization due to the conformation of the calcium phosphate on the alloy surface be three-dimensional. However, in the polished groups the cells presented a more elongated shape, emitting small extensions and flattened appearance, representing a more advanced stage of development in comparison to the treated groups.

From the third day, on all surfaces, the cells already emit the small prolongations and are in greater number, without preferential orientation. In the polished groups, after the eighth day, most cells are presented in polygonal format, united in single orientation, forming a cellular confluent which increased with the passage of time, at 15 days.

**Fig. 4:**
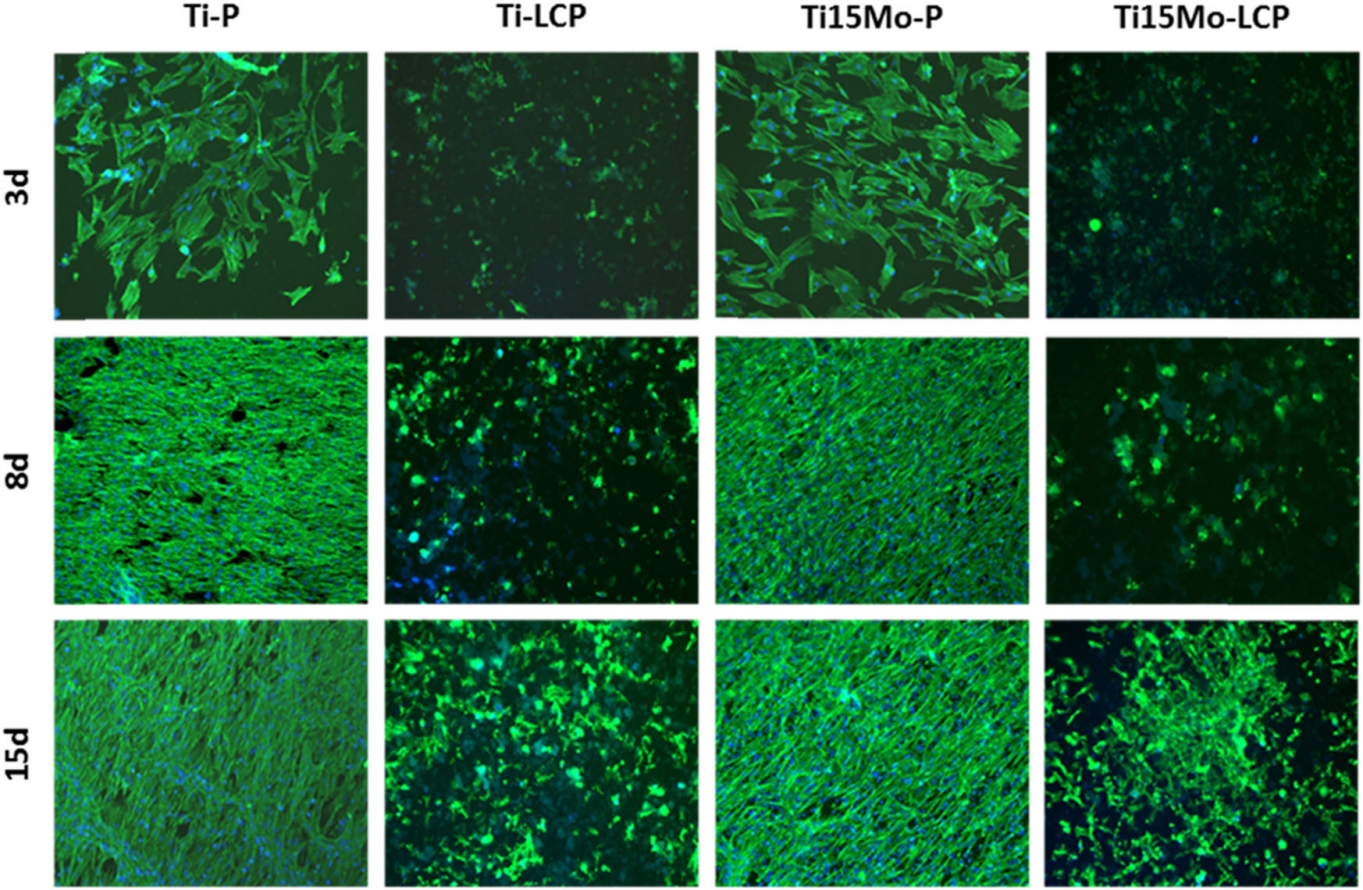
Cellular proliferation at 3, 8 and 15 days observed by direct fluorescence microscopy

In both Ti-LCP and Ti15Mo-LCP groups, it is only possible to visualize the cell proliferation, without showing its morphological stage due to the irregularities of the calcium phosphate structure (Fig. 4).

### Cellular viability

Cell viability was evaluated by the AlamarBlue^®^ test from 3 to 21 days of cell culture, being also an indirect indicator of proliferation based on the detection of cellular metabolic activity. Resazurin is reduced and transported out of the cells where it can be quantified by the spectrophotometer in the supernatant, and the number of viable cells is related to the level of dye reduction, whose viability is expressed as a percent reduction.

**Fig. 5:**
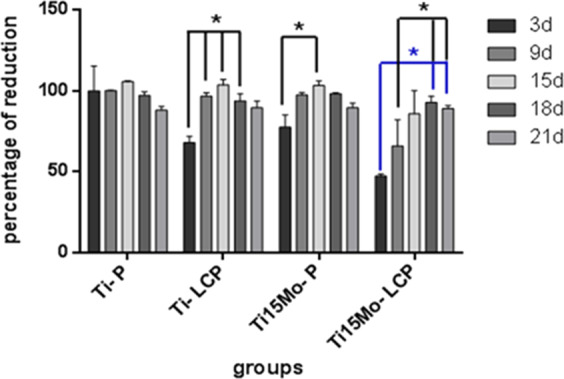
AlamarBlue^®^ - Percentage difference in reduction related to the positive control (%) - intra-groups comparison (*p < 0.05)

There was a statistical difference in the intra-group comparison in which the 3 days’ period had difference with 9, 15 and 18 days for the Ti-LCP group; 3 with 15 days for the Ti15MoP group; 3 days with 15, 18 and 21 days for Ti15Mo-LCP group, as well as 9 days with 18 and 21 days (*p* < 0.05). Despite the differences in the percentage of reduction in the initial periods of all groups, cell proliferation occurs over time, with its peak from 15 to 18 days. At 21 days, all groups presented similar percentages of reduction (Fig. 5).

### Alkaline phosphatase activity

**Fig. 6:**
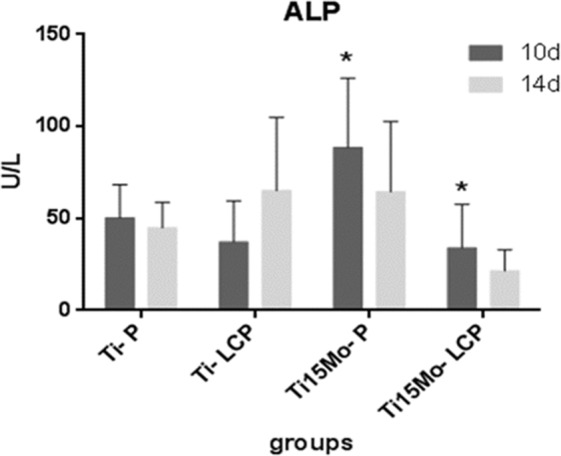
Alkaline phosphatase (ALP) production (U/L) at 10 and 14 days, for the different groups (Mean and SD; *p < 0.05)

The activity of ALP, an enzyme that plays a fundamental role in the mineralization of bone matrix with active osteoblasts, showed a higher activity in the 10-day period compared to the 14 days of culture, with a statistical difference in the Ti15Mo groups between P and LCP (*p* < 0.05) (Fig. 6).

### Gene expression of bone markers

To evaluate the maturation of cells in culture as well as the osteoblastic phenotype of the cells obtained from the bone marrow of rats and cultured in osteogenic medium, we analyzed several genes that encode proteins and transcription factors, associated with different maturation stages of osteoblasts. In general, no significant difference was observed for any gene and timepoint among the different tested surfaces. Some non-significant differences can be observed in the average of the expression; however, the variability of the results may have impaired the detection of statistically significant differences. The ALPL gene, which transcribed the enzyme alkaline phosphatase, was positively stimulated in cells cultured on the Ti-P group in comparison to the other groups, within 7 days. However, it was observed at 14 days maintenance of the stimulus for the cells of the Ti15Mo-P group, whereas in the other groups a decrease occurred in gene expression of ALPL.

**Fig. 7:**
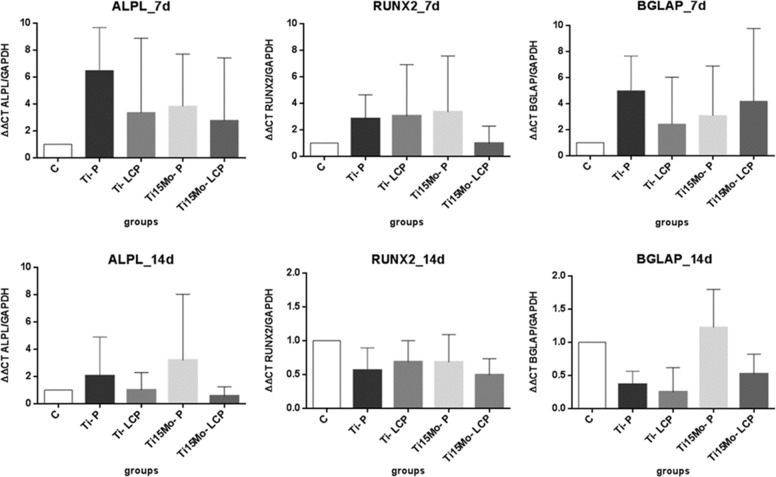
Alkaline phosphatase gene expression (ALPL), RUNX2 transcription factor and osteocalcin (BGLAP) at 7 and 14 days, for the different groups

The levels of gene expression of the RUNX2 transcription factor were lower for cells cultured in the Ti15Mo-LCP group compared to the other groups at 7 days of culture. At 14 days, expression levels were lower than the control, suggesting that the initial culture periods are more favorable for expression of this transcription factor. The relative expression of the mRNA of the BGLAP gene, also known as osteocalcin, was increased within 7 days of cell culture in cells seeded on the Ti-P and Ti15Mo-LCP groups. However, at 14 days, these levels remained for the Ti15Mo-P group and decreased for the other groups in relation to the control (Fig. 7).

## Discussion

The present study evaluated a new titanium alloy - Ti-15Mo - and the influence of its surface modification by laser-beam with calcium phosphate deposition on its physicochemical properties and the cellular behavior of primary osteoblast-like cells of rats cultured onto the samples. The cpTi was used as a control material. It has been shown that spray deposition of HA on the surface of dental implants can be partially dissolved after long periods of function [34, 35]. In addition, the plasma spray technique does not allow precise control of the chemical composition and the coating structure [34]. Thus, the biomimetic method has been considered one of the most promising technologies for surface modification of biomaterials and several studies have shown favorable results for Ti HA-coated, allowing a strong and stable layer of HA attached to the surface of the Ti implant [15, 16, 18, 32, 36].

According to Sisti et al. [37] laser irradiation associated with biomimetic deposition controls the thickness of the coated layer, enhancing the attachment of the HA to the metal substrate and improves surface topography increasing the host’s response to the implant optimizing the osseointegration.

Considering the development of Ti alloys with nontoxic elements and low elasticity modulus for biomedical applications [1, 2, 4, 8, 11], Oliveira et al. [12] showed that pulsed Yb:YAG laser treatment on Ti-15Mo alloy produced a typical rougher macro- and micro-structured surface, increasing surface wettability, bone-implant contact and removal torque measurements, improving Ti-15Mo implant osseointegration. Moreover, the mechanical properties of Ti and its alloys represent an important aspect for their clinical application. To minimize or avoid the stress shielding phenomenon (mismatch of Young’s moduli of the implant device and the bone) [4, 8, 11], β type Ti alloys (as Ti-15Mo used in this study) with low Young’s modulus (75 GPa - ASTM F2066-08), are desired compared to Ti and its alloys, Ti-6Al-4V, with a Young’s modulus around 110 GPa. Furthermore, it has been shown that neurological disorders (Alzheimer’s), allergic reaction and osteomalacia may be induced by the release of Vanadium (V) and Aluminum (Al) from Ti-6Al-4V alloys [9, 10].

In the present study, from the FEG-SEM analysis, it was possible verify that the laser irradiation and HA deposition to both cpTi and Ti-15Mo alloy groups, produced a unique 3D surface topography, in accordance to that found for cpTi obtained by Braga et al. [38], Heinrich et al. [39] and Queiroz et al. [18] and for Ti-15Mo alloy by Oliveira et al. [12], Guastaldi et al. [40] and Pires et al. [13].

The literature reports several studies that employs the technology of laser modification of cpTi and Ti alloys, with improvements in their physicochemical properties, wear and corrosion resistance, favoring the in vivo bone response to these surfaces [12, 13, 15, 16, 18, 40]. To our knowledge this is the first study that combines laser surface modification of this new Ti-15Mo alloy and HA deposition on the behavior of osteoblast-like cells.

After implant surface modification, a key step relies on the possible chemical contamination of the surface. From the EDX analysis, it was possible to prove that no contamination was found after laser irradiation and HA deposition to both cpTi and Ti-15Mo alloy groups. Similar findings were observed by Bini et al. [15] and Filho et al. [16] after laser irradiation on cpTi surface, Oliveira et al. [12, 41], Guastaldi et al. [40] and Pires et al. [13] after laser irradiation on Ti-15Mo alloy surface and Queiroz et al. [18] after laser irradiation and HA deposition on cpTi surface. In addition, using X-ray diffraction (XRD) and Fourier-transform infrared spectroscopy (FTIR) analysis, it has already been shown the phases and the chemical composition of titanium samples (cpTi and Ti-15Mo) after laser surface modification and deposition of calcium phosphates using the same methodology described in this manuscript (Bini et al. [15]; Filho et al. [16]; Santos et al. [42–44]).

Regarding the results of the contact angle assay, a complete wetting for the laser and HA deposition groups was shown. Similar results were observed by Oliveira et al. [12, 41], Guastaldi et al. [40] and Pires et al. [13] after laser irradiation on Ti-15Mo surface and Queiroz et al. [18] after laser irradiation and HA deposition on cpTi surface. It is known that osteoblast culture exhibits better adhesion and growth on hydrophilic surfaces [45] and wettability influences the osteoblastic response in vitro [46].

Hydrophilicity is considered an important factor to early bone response [47, 48], which can accelerate the healing process and consequently shorten the time of loading protocols [49]. It has been shown that both higher surface energy and increased wettability properties directly influences the interactions between a surface and its biologic environment [50]. After the placement of a dental implant into bone, the surface is exposed to tissue fluids, producing a layer of macromolecules and fluids, influencing the behavior of cells when they encounter the implant surface [51].

Using osteoblasts and biomaterials to study cell growth, Ramires et al. [52] showed that there was early cellular aggregation, cell differentiation and mineralization on HA-coated TiO2 surfaces. Other studies have also shown that apatite coating may facilitate osteogenic differentiation and promote bone growth [53–55]. The results of the present study demonstrated that cells grown onto polished Ti and Ti15Mo samples showed similar spreading for both groups, in which cells were rounded and adhered to the surface at 3 days and in high number and more extensions, forming agglomerates at 8 days of cultivation. On LCP treated surfaces, the treatment appears to favor cell growth compared to P surfaces. There is an increase in the specific surface area for cell contact created by the laser irradiation and the HA structure in which the cellular extensions attach, forming a three-dimensional cellular structure, similar to osteoblasts, as well as in the studies of Zhu et al. [56] in which the incorporation of BMP-2 within the calcium phosphate coating facilitated the spread of osteoblasts.

Both substrates and surfaces allowed proliferation and maintenance of viable cells, similarly, throughout the experiment (21 days). The HA-coated surfaces, regardless the substrates, showed a tendency to lower cellular amount at 3 days, but with rapid proliferation after this period, matching the other groups in later periods.

The influence of the proposed surface modification on the gene expression of cells was demonstrated by real-time PCR analysis. Although no statistically significant difference was found between the groups, the average expression results suggest that P surfaces had a greater influence on the expression of the ALP gene, the RUNX2 transcription factor and the BGLAP gene mRNA at 7 days of cell culture, which may be related to a larger number of cells in these groups in the initial periods. The gene encoding alkaline phosphatase enzyme is essential during the stages of mineralization performed by osteoblastic cells, as an indication of calcification and differentiation, as RUNX2 expression increases during cell differentiation, validating its involvement in osteoblast maturation [57, 58]. However, at 14 days, these levels decreased for all groups. Oliveira et al. [41] showed in laser-treated Ti-15Mo alloy disks, that the expressions of RUNX2, ALPL and SPP1 remained unchanged compared to the cpTi polished disks.

Cells grown on roughened surfaces tend to exhibit greater osteoblastic differentiation compared to those grown on smoothed surfaces, fewer cells and usually increased alkaline phosphatase activity [59]. The ALP activities on day 10 were higher than day 14, with a statistical difference in the Ti-15Mo alloy between P and LCP (*p* < 0.05) probably due to the lower proliferation of the LCP group, compared to the P surface, in the initial periods, as observed in the cell viability and proliferation tests.

The complex 3D morphology of the laser irradiated, and HA-coated surface may have impaired the proper analysis and adequate interpretation of the images regarding cellular morphology by the bi-dimensional fluorescence analysis. Additional three-dimensional analysis needs to be performed in the future to confirm proliferation results from the AlamarBlue^®^ assay. Also in vivo studies are needed to evaluate the bone response to this new Ti alloy with laser-beam surface modification and biomimetic HA deposition for biomedical applications.

From a clinical perspective, the use of titanium alloys with low Young’s modulus, composed of biocompatible and nontoxic elements, such as Ti-15Mo alloy, might represent an important alternative to cpTi and Ti-6Al-4Va alloy for the manufacture of dental implants. In addition, laser-beam surface modification followed by calcium phosphate of biological interest deposition have shown to be promising and economically feasible and may improve and accelerate the osseointegration process of dental implants reducing the overall treatment time.

## Conclusions

The laser parameters employed associated with the biomimetic deposition of HA created a unique 3D surface that proved to be a controllable, reproducible and a clean method to modify cpTi and Ti-15Mo alloy surfaces. Comparing the biological mechanism of the interaction of osteoblast-like cells with the employed surfaces, it can be concluded that the Ti-15Mo alloy does not interfere in the behavior of these cells and showed similar behavior to Ti groups.
